# Leukemoid Reaction in Infant Pertussis: Is There a Place for Hydroxyurea? A Case Report

**DOI:** 10.3389/fped.2018.00261

**Published:** 2018-09-26

**Authors:** Guillaume Maitre, Damien Schaffner, Julia Natterer, David Longchamp, Thomas Ferry, Manuel Diezi, Stefano Di Bernardo, Marie-Hélène Perez, Vivianne Amiet

**Affiliations:** ^1^Pediatric Intensive Care Unit, Lausanne University Hospital, Lausanne, Switzerland; ^2^Pediatric Onco-Hematology Unit, Lausanne University Hospital, Lausanne, Switzerland; ^3^Pediatric Cardiology Unit, Lausanne University Hospital, Lausanne, Switzerland

**Keywords:** bordetella pertussis, hydroxyurea, hyperleukocytosis, leukemoid reaction, white blood cell count

## Abstract

A 73-days old infant of 34 weeks' gestation was hospitalized with a co-infection of respiratory syncytial virus (RSV) and *Bordetella pertussis (BP)*. She required invasive ventilation for 9 days in the context of malignant pertussis with persistent hypoxemia and hypercapnia secondary to a leukemoid reaction. Despite an increase of white blood cell (WBC) count up to 70 G/L and ensuing pulmonary hypertension, no hemodynamic compromise occurred. Without clear indication for leukapheresis nor exchange transfusion, an off-label treatment with hydroxyurea was given for 5 days with gradual decrease of WBC count, without any complication and hospital discharge on day 29. To our knowledge, no effective therapy for malignant pertussis has been described in the literature and complications are frequent with leukoreduction procedures. We discuss an alternative to invasive procedures in young infants to fulfill the need to decrease rapidly leukocyte counts in a leukemoid reaction associated with *Bordetella pertussis* infection. To our knowledge, hydroxyurea has never been used in malignant pertussis but is a well-known medication for oncologic and hematologic diseases such as acute myeloid leukemia or sickle cell anemia. Its effects in this setting are not well understood but the positive outcome in our patient supports the need for further studies.

## Background

*Bordetella pertussis* is a worldwide, human-restricted bacterium that was first isolated in 1906 by Jules Bordet. The World Health Organization (WHO) estimates that this pathogen was found in 16 million infectious disease worldwide and caused 195,000 deaths in 2008, 95% of which occurred in developing countries (1). In Switzerland, between 2010 and 2014, about 8,700 annual cases were identified (declaration is mandatory in our country) despite the recommended national vaccination program. Currently, about 30 children are hospitalized each year, mainly infants, and four pertussis related deaths have been reported in the past 15 years (2).

Malignant pertussis, the most severe form, is characterized by major leukocytosis, refractory hypoxemia, pulmonary hypertension and cardiopulmonary compromise, with high morbidity and mortality. A prospective study in the United States showed a 10-fold increase in the risk of death in the presence of leukocytosis >50 G/L. There was also a clear association between high median white blood count, mechanical ventilation and pulmonary hypertension (3). In this cohort, the majority of the patients was less than 3-month-old.

Leukocytosis causes a hyperviscosity syndrome with leukostasis, which can be complicated by intracranial hemorrhages and pulmonary hypertension (4). Targeted antibiotic therapy is the first line treatment, in combination with supportive care (invasive ventilation, oxygenation, nutritional support). Leukapheresis or exchange transfusion are adjunctive therapies proposed to reduce leukocytosis and its adverse consequences (4–6).

Hydroxyurea has been used to reduce blood counts in several oncologic and hematologic disorders such as acute myeloid leukemia, polycythemia, thrombocythemia and sickle cell disease. To our knowledge, hydroxyurea has never been used in malignant pertussis. We report the case of an infant with malignant pertussis and leukemoid reaction treated by hydroxyurea.

## Case presentation

Seventy-three days-old infant born prematurely at 34 1/7 weeks of gestation with a birth-weight of 1,765 g (P 10-25) was admitted for respiratory syncytial virus bronchiolitis with runny nose and decreased food intake. She was vaccinated according to Swiss recommendations (Infanrix pentavalent®/Prevenar®) at 2-months of age. Her parents were not vaccinated against *BP*.

On day 4 of hospitalization, she developed severe respiratory distress. Chest X-ray showed a right upper lobe pulmonary infiltrate. Blood workup revealed an elevated C - reactive protein (CRP 210 mg/L) and hyperleukocytosis (64 G/L) (differential blood count initially not done). Due to clinical respiratory distress and hypercapnia, non-invasive ventilation was started. Hypercapnia worsened despite this treatment, and the child needed mechanical ventilation and was transferred to our Pediatric Intensive Care Unit (PICU). Due to the respiratory worsening associated with increasing inflammatory parameters, a bacterial infection was suspected and intravenous antibiotic treatment with ceftriaxone 100 mg/kg/day was started. Subsequent workup showed a positive *Streptococcus pneumoniae* antigen in urine and a positive *Bordetella pertussis* PCR in the nasal sample. *BP* had been specifically searched due to the severe hyperleukocytosis associated with lymphocytosis and intravenous clarithromycin 20 mg/kg/day was added to ceftriaxone.

**Figure 1:**
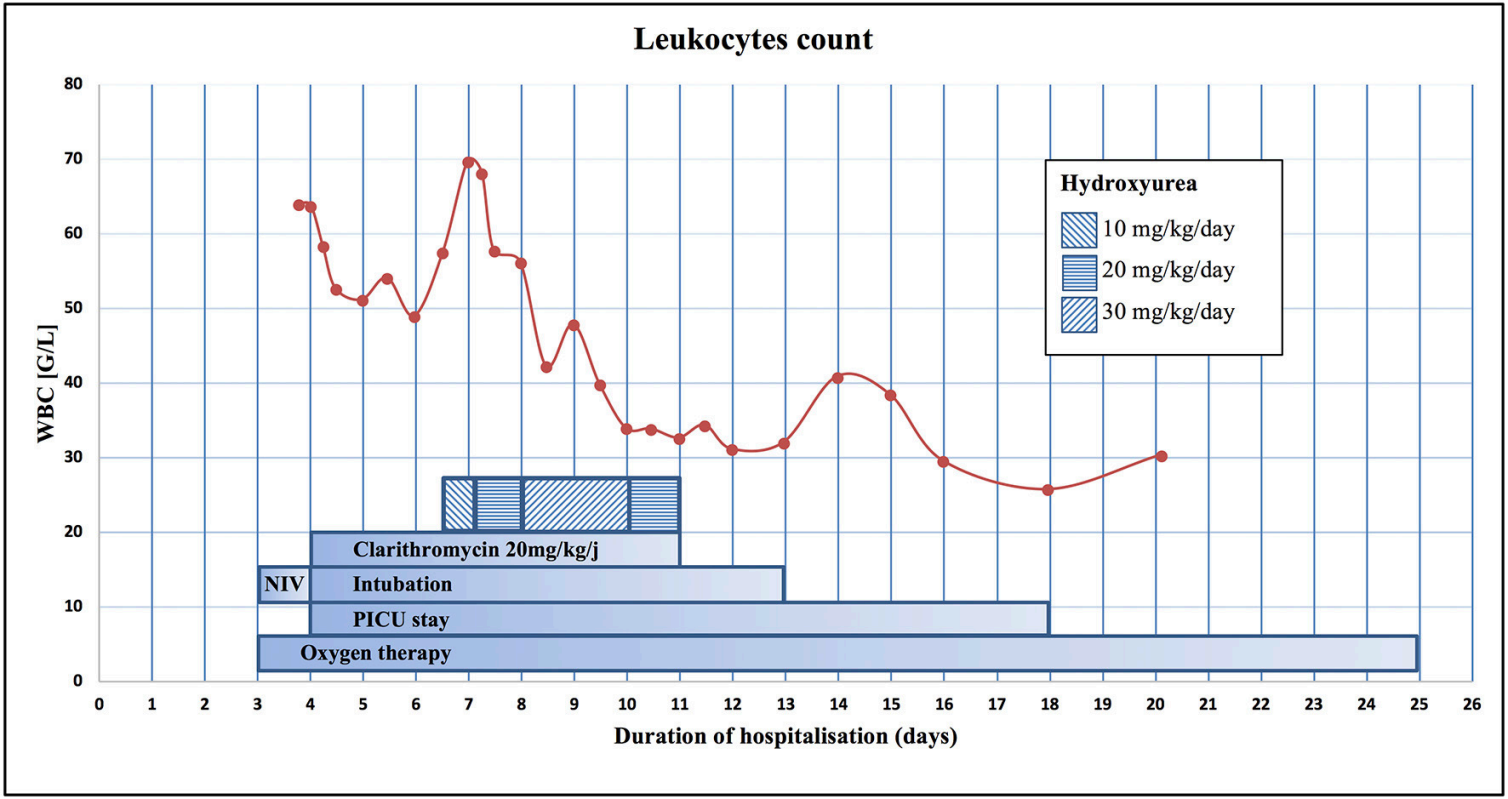
Evolution of WBC during the hospitalization and representation of the different therapies. NIV, Non-Invasive Ventilation.

The child required high ventilatory parameters and up to 100% oxygen. Despite this ventilatory support, hypercapnia persisted. An echocardiogram showed pulmonary hypertension but a preserved right ventricular function. Leukocytosis first decreased probably due to fluid resuscitation and consecutive haemodilution. On day 6, leukocytosis worsened (Figure 1) but leukapheresis was not yet clearly indicated. As respiratory status was not improving, we decided to introduce hydroxyurea (Litalir®) at an initial dosage of 10 mg/kg/day. This treatment was increased gradually up to 30 mg/kg/day with a subsequent decrease in leucocytes count (Figure 1). On day 10, leucocytes decreased to 34 G/L and hydroxyurea treatment was discontinued to avoid iatrogenic leukopenia (Table 1). Simultaneously, respiratory condition improved, and the child could be extubated on day 13. Both antibiotics were stopped after 7 days of treatment. Oxygen therapy was required until day 25. The child was discharged home on day 29 without any complications.

**Table 1:** Differential blood count and platelets during PICU stay.

	**Leukocytes G/L**	**Lymphocytes G/L (%)**	**Neutrophils G/L (%)**	**Bands G/L (%)**	**Monocytes G/L (%)**	**Eosinophils G/L (%)**	**Basophils G/L (%)**	**Platelets G/L**
Day 4	63.8	28.7 (45)	30.6 (48)	6.4 (10)	1.3 (2)	0.6 (1)	0.0 (0)	874
Day 5	51.2	25.6 (50)	20.0 (39)	0.0 (0)	3.1 (6)	0.0 (0)	0.5 (1)	857
Day 6	49.0	25.5 (52)	21.1 (43)	0.0 (0)	1.5 (3)	0.0 (0)	0.0 (0)	892
Day 7	69.7	30.7 (44)	35.5 (51)	1.4 (2)	2.8 (4)	0.7 (1)	0.0 (0)	895
Day 8	42.3	20.7 (49)	19.0 (45)	0.0 (0)	2.1 (5)	0.4 (1)	0.0 (0)	701
Day 9	47.9	23.5 (49)	22.5 (47)	0.0 (0)	1.4 (3)	0.0 (0)	0.0 (0)	739
Day 10	33.9	18.6 (55)	13.6 (40)	0.3 (1)	1.7 (5)	0.3 (1)	0.0 (0)	656
Day 11	32.7	18.0 (55)	10.8 (33)	0.0 (0)	1.3 (4)	0.3 (1)	0.0 (0)	627
Day 12	31.2	19.0 (61)	8.7 (28)	0.0 (0)	2.2 (7)	0.9 (3)	0.0 (0)	597
Day 13	32.1	19.3 (60)	11.2 (35)	0.6 (2)	1.3 (4)	0.0 (0)	0.0 (0)	628
Day 14	40.9	22.1 (54)	16.8 (41)	0.0 (0)	1.6 (4)	0.4 (1)	0.0 (0)	664
Day 15	38.5	15.4 (40)	19.3 (50)	0.0 (0)	3.9 (10)	0.0 (0)	0.0 (0)	648
Day 18	25.8	11.9 (46)	11.4 (44)	0.0 (0)	1.8 (7)	0.8 (3)	0.0 (0)	651

## Discussion

### Pertussis and leukemoid reaction

This patient presented with a severe form of malignant pertussis and secondary leukemoid reaction. This hematologic reaction, occurring in the context of *BP* infection, was first described more than a century ago and has a diagnostic value since 1898 (7). Infants typically show this kind of reaction characterized by WBC count over 50 G/L with a marked lymphocytosis. This reaction is different from hyperleukocytosis occurring specifically in an oncological context and defined by WBC count greater than 100 G/L in the peripheral blood (8).

Leukemoid reaction is caused by *BP* toxin, but the specific mechanisms are not yet clearly understood. One of the proposed mechanisms might be the inhibition of lymphocyte extravasation to the infected site and facilitation of lymphocyte migration from the spleen and the bone marrow, resulting in leukocytosis. Phenotypic analyses show that pertussis leukocytosis consists of an expansion of normal naïve cells population rather than proliferation of activated cells (7). Another effect of pertussis toxin is the inactivation of many G proteins involved in cellular regulatory mechanisms. Some of these G proteins have protective effects on the cardiorespiratory system and their inactivation explains the rapid increase in heart and respiratory rate seen in these situations. One hypothesis is that leukocytosis is the marker of toxin activity, and inhibition of G proteins in the heart and lung may be the cause of death (9).

Several studies have shown a significant association between leukemoid reaction and fatal evolution, either regarding the absolute value of leukocytosis or its rate of progression (3, 9–14). Although most agree that poor prognosis is highly correlated with leukocytosis >100 G/L, a negative impact on survival of leukocyte counts >55 G/L has also been shown (15). Other factors of poor prognosis identified are low birth weight, gestational age, seizure during infection and pulmonary hypertension (3). The patient we describe meets several of these factors, namely: low birth weight, prematurity and pulmonary arterial hypertension.

### Urgent need to decrease leukocytes count

Hyperviscosity and leukostasis induced by the increase of circulating leucocytes leads to potential major complications such as intracranial bleeding and pulmonary failure with hypoxia and hypercapnia. Most post-mortem analyses reveal the presence of thrombi in venous, arterial and lymphatic pulmonary vessels (4, 16–19). In this context, the development of pulmonary arterial hypertension seems to be multifactorial: increased pulmonary resistance due to hyperviscosity in the setting of leukocytosis and thrombi; vasoconstriction induced by hypoxia in young children whose pulmonary vascular reactivity is increased; and, as mentioned above, the direct effect of the toxin on heart and lungs (14, 19). Acting on leukocytosis is essential to limit the hemodynamic consequences and the use of cardiac and respiratory support, such as extra-corporeal membrane oxygenation (ECMO), which has a high mortality rate without concomitant leukoreduction.

Two way of managing hyperleukocytosis, beside chemotherapy, are currently used for urgent cytoreduction in severe or symptomatic cases: leukapheresis and exchange transfusion. Leukapheresis is the treatment of choice in cases of hyperviscosity with hemodynamic impact, but there are some limitations of this technique: difficult vascular access in young children, risks associated with anticoagulation, poor experience or unavailability of the procedure (8). In a case series of 102 children with acute leukemia and hyperleukocytosis treated with leukapheresis, Abla et al. described a complication rate of 86%. Most complications in this study were metabolic disorders, hemodynamic instability and coagulopathy (20). Exchange transfusion is a useful and safer alternative, since it is widely used for other diseases. The complications are the same as for any transfusions (4). Several studies have shown a quick improvement of hypoxemia following a leukodepletion therapy in pertussis infection, whether by leukapheresis or exchange transfusion. The study of Rowlands et al. demonstrated the benefit of fast leukoreduction strategy in 10 patients younger than 3 months old with hyperviscosity syndrome during pertussis infection and with consecutive cardiovascular, pulmonary and neurological repercussions (21). Between 2005 and 2009, five patients underwent leukapheresis under ECMO while five others only exchange transfusion. The global survival rate was 90%. A similar study between 2001 and 2004 of 9 patients, including 6 treated with ECMO, without any leukocyte reduction technique, showed survival of 55% (21). Several other small case series reporting experience of exchange transfusion in malignant pertussis showed survival rates between 54 and 92% (4, 22–25). Some authors point out that exchange transfusion should be preferred over leukodepletion methods because it simultaneously removes pertussis toxin (9).

### An alternative to invasive procedures?

Our patient had a respiratory failure with refractory hypoxemia and hypercapnia, despite a high ventilatory support, pulmonary hypertension without cardiac repercussion and no neurologic symptoms. Even if the WBC count increased in a worrying way, it remains under 100 G/L. In the absence of clear criteria in the literature to arbitrate for leukapheresis and considering the risks of this procedure, we sought an alternative therapy to reduce leukocytosis. By analogy with its use in oncological context, an off-label treatment with hydroxyurea (Litalir®) was introduced.

Hydroxyurea, or hydroxycarbamide, is an old drug, whose inhibitory effects on leucocytes is known since the end of the 1860s. Since the 1960s, the efficacy of hydroxyurea on hyperleukocytosis associated with acute myeloid leukemia is well described, especially on the rapid reduction of blast cells, thus preventing the complications of hyperleukocytosis (26, 27). Its use has spread to other types of cancer (carcinoma of the head or the neck), and to some non-neoplastic pathologies such as HIV infection or sickle cell anemia. Hydroxyurea is an antiproliferative medication interfering with DNA synthesis. The cytostatic effect is driven by ribonucleotide reductase inhibition and thus cell cycle arrest in the absence of sufficient nucleotide pools (28). Absorption through the digestive system is rapid with plasma peaks reached within 1 h. Hydroxyurea accumulates particularly in blood cells, especially the white line. The passage through the blood-brain barrier is easy. Entry into cells is a passive phenomenon with rapid balancing of concentration between different tissues and blood (28, 29).

In the sickle cell anemia, hydroxyurea improves the pathophysiology and seems to be the only treatment with proven benefits. This treatment reduces acute complications, such as painful vaso-occlusive events and acute thoracic syndrome, as well as need for blood transfusions and hospitalizations. The survival in adult and child is also improved (30). In this case, hydroxyurea increases production of fetal hemoglobin via nitric oxyde production and decreases adherence of red blood cell to the vascular endothelium (31).

In HIV infection, hydroxyurea has an inhibitory effect on viral replication. Benito et al. studied the effect *in vitro* on T cell proliferation and activation. A dose-dependent effect on the proliferation of T cells, with a decreased average number of mitoses was shown. An impact on the expression of the different markers of T cell activation was also demonstrated (32).

This mechanism of action may explain the potential benefit of hydroxyurea to decrease lymphocytosis in whooping cough, which mainly increases lymphocytes. Nevertheless, as previously discussed, leukocytosis in pertussis is an expansion of naive cells and not a proliferation of new cells. Another mechanism causing the cell-killing effect of hydroxyurea would be the production of oxidative stress and the induction of a cessation of cytokinesis, as some studies seem to show (31). None of the known mechanisms of action of hydroxyurea alone seems to counteract the effects of pertussis toxin, but many of these mechanisms may be involved. Although the action of hydroxyurea on leukocytosis caused by the pertussis toxin is not yet understood, its effect in our patient was positive. We found a resolution of the leukemoid reaction after 5 days of treatment without developing lysis syndrome or other recognizable adverse reactions. We hypothesize that part of leukocytosis seen in malignant pertussis may be due to overproduction of new white cells which is then decreased with hydroxyurea treatment. Nevertheless, given that 50% patients spontaneously recover with supportive therapy alone, the current outcome of the patient maybe is not the consequence of hydroxyurea therapy.

The speculative risk of hydroxyurea treatment is the consecutive myelosuppresion, leukopenia, thrombocytopenia and anemia but there is no clear evidence in the literature of severe complications with standard dosage (between 10 and 30 mg/kg/day).

New experimental therapies, such as monoclonal antibodies against pertussis toxin or other targets to reduce pulmonary damage are under investigation. These approaches seem to be promising on mice models but need further evaluation (33, 34).

## Concluding remarks

Currently there is no effective treatment for malignant pertussis. Management of these patients is mainly supportive. Leukapheresis, exchange transfusion and ECMO therapy have been associated with high mortality and complications (17). Malignant pertussis is an old, well-known disease and perhaps an old drug like hydroxyurea may be a new treatment strategy in this setting. This non-invasive approach needs further studies, but hydroxyurea used preventively may avoid heart failure or neurological symptomatology and the subsequent need of for invasive leukoreduction procedures and associated complications.

## Ethics statement

Parents gave their written informed consent to publish this case report.

## Author contributions

VA and M-HP conceptualized the work. GM and DS analyzed the data. GM and DS drafted and JN, DL, TF, MD, SD, M-HP, and VA revised this manuscript. All authors approved the final version and agreed to be accountable for the content of the work.

### Conflict of interest statement

The authors declare that the research was conducted in the absence of any commercial or financial relationships that could be construed as a potential conflict of interest.

## Abbreviations

*BP*: *Bordetella pertussis*
WBC: white blood cell
ECMO: extracorporeal membrane oxygenation
RSV: respiratory syncytial virus
PICU: pediatric intensive care unit.
